# Plasma and Cerebrospinal Fluid Concentrations of Micafungin Administered at High Doses in Critically Ill Infants with Systemic Candidiasis: A Pooled Analysis of Two Studies

**DOI:** 10.3390/ph16030472

**Published:** 2023-03-22

**Authors:** Domenico Umberto De Rose, Iliana Bersani, Maria Paola Ronchetti, Fiammetta Piersigilli, Sara Cairoli, Andrea Dotta, Amit Desai, Laura Lynn Kovanda, Bianca Maria Goffredo, Cinzia Auriti

**Affiliations:** 1 Neonatal Intensive Care Unit, “Bambino Gesù” Children’s Hospital IRCCS, 00165 Rome, Italy; iliana.bersani@opbg.net (I.B.); mariapaola.ronchetti@opbg.net (M.P.R.); andrea.dotta@opbg.net (A.D.); cinzia.auriti@opbg.net (C.A.); 2 Section of Neonatology, Cliniques Universitaires Saint Luc, Université Catholique de Louvain, 1200 Brussels, Belgium; 3 Biochemistry Laboratory, “Bambino Gesù” Children’s Hospital IRCCS, 00165 Rome, Italy; sara.cairoli@opbg.net (S.C.); biancamaria.goffredo@opbg.net (B.M.G.); 4 Astellas Pharma Global Development Inc., Northbrook, IL 60062, USA; amit.desai@astellas.com (A.D.); laura.kovanda@astellas.com (L.L.K.)

**Keywords:** therapeutic drug monitoring, drug doses, fungal infections, antifungals

## Abstract

**Background**: Neonates may require higher doses of micafungin than adults to reach the therapeutic effect for increased plasma clearance. Only poor and inconclusive data are available still now to support this hypothesis, especially with regard to central nervous system micafungin concentrations. To assess the pharmacokinetics of increased doses (8 to 15 mg/kg/day) of micafungin in preterm and term neonates with invasive candidiasis and to complete previously presented results, we analyzed the pharmacokinetic data on a total of 53 newborns treated with micafungin, whereby 3 of them had *Candida* meningitis and hydrocephalus. **Methods**: Fifty-three neonates with systemic candidiasis, three of them with meningitis, were treated for at least 14 days with intravenous micafungin (Mycamine^®^) at a dosage ranging from 8 to 15 mg/kg/day. Plasma and cerebrospinal fluid (CSF) concentrations of micafungin were measured before the drug administration and at 1, 2, and 8 h after the end of the infusion using high-performance liquid chromatography (HPLC). Systemic exposure was assessed according to AUC_0–24_, plasma clearance (CL), and half-life measured in 52/53 patients, divided by chronological age. **Results and conclusions**: The mean micafungin clearance is higher in neonates than in older infants (0.036 L/h/kg before 28 days of life versus 0.028 L/h/kg after 120 days). The drug half-life is shorter in neonates than in older patients (13.5 h before 28 days of life versus 14.4 h after 120 days). With doses ranging between 8 and 15 mg/kg/day, micafungin crosses the blood–brain barrier reaching therapeutic levels in CSF.

## 1. Introduction

Micafungin is an antifungal which selectively inhibits the enzyme beta 1,3 glucan synthase, blocking the synthesis of 1,3-b-D-glucan (one of the essential components of the fungal cell wall) and causing lysis of the fungal cell. This mechanism of action explains their characteristics of high specificity, even against resistant species of *Candida*. Micafungin is primarily eliminated via biliary excretion (90%), while the route of elimination via the kidney appears to be of minor importance in humans. Micafungin is bound to plasma proteins up to 99.5%. The binding to plasma proteins seems to be about eight times lower in newborns than in adults, with an increase in the proportion of the free drug that could be rapidly eliminated [1]. In Europe, micafungin is authorized in the treatment of systemic candidiasis of the newborn, but the literature still reports deep uncertainty about the drug pharmacokinetics (PKs) and the recommendable dose to reach therapeutic exposure in these delicate patients. It has been suggested that infants require higher doses of micafungin than adults to achieve a therapeutic effect due to increased plasma clearance of the drug [2,3]. The functional development of organs such as the liver and kidney is in progress in neonates, and the possibility of changes in distribution, metabolism, and excretion should be considered for drugs administered intravenously. Neonates, especially preterm neonates, have lower body fat storage and higher total body water (TBW) than children and adults [4,5]. These factors significantly influence the distribution volume of drugs in newborns. The protein binding of drugs also determines their dispersion throughout the body. Most plasma-binding proteins (e.g., albumin, 1-acid glycoprotein, or plasma globulins) are lower in newborns than adults, and low albumin levels contribute to a higher elimination of drugs. Additionally, in infants, there may be an increase in bilirubin and free fatty acid concentrations, which may result in a competitive binding of the drugs to albumin [6,7].

Yanni et al. observed an increased clearance of micafungin in infants and children: 40–80 mL/h/kg in premature infants and 20 mL/h/kg in children >4 months of age. While they evidenced no differences in intrinsic hepatobiliary clearance of micafungin between neonates and adults, their data suggested that the age-dependent serum protein binding of micafungin may be responsible for increased drug elimination in infants [1].

Hope et al., in order to better understand the mechanisms of micafungin clearance, constructed a two-compartment model, incorporating body weight as a predefined covariate for the allometric scaling of the pharmacokinetic parameters and identifying aspartate aminotransferase and total bilirubin as covariates that had a significant effect on micafungin clearance. They concluded that clearance in smaller children is higher than that predicted on the basis of weight alone [8].

Moreover, the high risk of early neurological localization in systemic candidiasis of preterm infants requires a dosage of antifungals that guarantees passage through the blood–brain barrier with certainty. The lack of information on the optimal dosage to resolve such infections, together with the difficulty in carrying out repeated CSF dosages in neonates, has led to consensus among experts. Therefore, the doses of micafungin currently used range between 4 and 10 mg/kg in premature infants and between 2 and 4 mg/kg in older children with invasive candidiasis, but micafungin is still not recommended as the first option in neonates with systemic candidiasis [3,9] due to this uncertainty about the most appropriate dose.

To better explore the efficacy and the safety of doses that were 4- to 7.5-fold higher than those approved by regulatory agencies for use in neonates with invasive candidiasis, we performed two studies in both preterm and term infants with fungal infections, until the age of six months [10,11]. In the current report, we present a pooled analysis of the pharmacokinetics data on the total of 53 neonates treated with micafungin in the previous two studies, in order to provide a complete overview of our results.

## 2. Materials and Methods

### 2.1. Design of the Study

Two previous studies were carried out at the Neonatal Intensive Care Unit of Bambino Gesù Hospital in Rome: the first retrospective study was on data collected from December 2012 to April 2015 on 18 patients; the second prospective study was on patients enrolled from May 2015 to September 2018 on 35 patients. The drug administration protocol, timing of blood draw, and analysis procedure of the collected samples were the same in the two studies.

For this pooled analysis, we included data from a total of fifty-three neonates enrolled, aged ≤180 days (from the gestational age corrected at 37 weeks), affected with proven or suspected systemic candidiasis, whereby 3 of them had meningitis, who were treated for at least 14 days with micafungin (Mycamine^®^) at a dosage ranging from 8 to 15 mg/kg/day intravenously. 

The dosage was formulated as a 2 mg/mL solution in 0.9% saline solution and administered intravenously over 1 h once daily. Infants who died within the first 48 h and those with acute or chronic hepatic diseases and/or known hypersensitivity to echinocandins were excluded. Each study with specific inclusion/exclusion criteria and definitions has been described in detail elsewhere [10,11]. 

Micafungin plasma and CSF concentrations were measured 1 h before and 1, 2, and 8 h after the end of the infusion. Sampling overlapped with those required for the clinical monitoring of these critical children. Blood samples were collected via heel puncture, using 0.3 mL capillary microtubes with EDTA, between Days 3 and 10 of micafungin therapy. Five minutes prior to sampling, the heel was warmed with a hot compress (40 °C), and puncture was performed using a Gentleheel incision device (Cardinal Health, Dublin, OH, USA) at alternate sites per timepoint. Blood picked up was centrifuged at 3000× *g*, and the plasma residue was stored at 4 °C until analyzed within 6 h or frozen at −20 °C and analyzed within 7 days, as previously reported [12]. Drug concentrations were measured via high-performance liquid chromatography (HPLC). An HPLC method with a diode array detector was validated to fine-tune the measurement on small amounts of plasma (0.3 mL whole blood) [13,14].

### 2.2. Outcomes

We aimed to assess plasmatic and CSF levels of micafungin administered intravenously at high dosages (8–15 mg/kg/day) in neonates and small infants.

Moreover, we assessed the optimal dosage to obtain systemic drug exposure equivalent to that considered to be effective in adult patients. Systemic drug exposure was assessed via the area under the curve for 24 h (AUC_0–24_), the plasma clearance (CL), and the half-life. Results were stratified according to chronological age. 

In order to evaluate drug safety, we analyzed treatment-emergent adverse effects (TEAEs). TEAEs were defined as an adverse effect experienced any time during the study’s drug administration to 72 h after the last dose of the study drug. A reasonable possibility that the event could have been caused by the study drug was assessed by the investigators; if the relationship was missing, it was considered to be drug-related.

### 2.3. Ethical Approval

The current study was conducted in accordance with the standard of good clinical practice, the principles expressed in the Declaration of Helsinki, and all applicable local regulations.

Concerning two previous studies, the Institutional Review Board of “Bambino Gesù” Children’s Hospital (protocol code 800_OPBG_2014, approved on 12 November 2014, and protocol code 978-OPBG_2015, approved on 29 July 2015) examined and approved the two protocols. These protocols were initially designed as non-profit academic studies and on 5 December 2017, the Italian Medicines Agency approved the transfer of the sponsorship of the protocol of the second study from Bambino Gesù Children’s Hospital to Astellas company. The trial was registered on http://www.clinicaltrials.gov (NCT03421002) (accessed on 16 February 2023). However, the funder had no role in designing the studies, fine-tuning the technique for carrying out the PK assays, carrying out the laboratory measurements, monitoring the patients during therapy, checking the laboratory tests, collecting data, collecting parental consent from enrolled patients, or checking the results. 

Written informed consent was obtained from the parents of all infants involved in the study before conducting any procedures and, concerning the second study, upon sponsor transfer to Astellas. The U.S. Food and Drug Administration also conducted a Biological Research Monitoring Inspection (BIMO) in October 2019 to determine whether the hospital and clinical research operations were being conducted in compliance with regulations on the current good clinical practice (GCP) for the study: the outcome of the inspection was very favorable.

### 2.4. Statistical Analysis

Descriptive statistics were used to characterize the demographic and baseline laboratory parameters. Data are presented as numbers and percentages for categorical variables. Continuous variables are expressed as the mean ± standard deviation (SD) if they are normally distributed or as the median and interquartile range (IQR) if normality could not be accepted. The probability of target attainment (PTA) was estimated for different dosing regimens via Monte Carlo analysis in relation to the identified minimum inhibitory concentrations (MICs); the target range of an AUC of at least 170 µg h/mL was derived from a previous experimental hematogenous *Candida* meningoencephalitis model [1]. 

All statistical analyses were performed via the use of the Stata SE, v. 13.1, program (StataCorp LP, College Station, TX, USA). 

## 3. Results

From December 2012 to September 2018, fifty-three infants with proven or probable systemic candidiasis were treated with micafungin. Of these, three infants had fungal meningitis and hydrocephalus drained via external shunt. Thirty-three infants (62.3%) were males. The median birth weight was 1.30 kg (IQR: 2.00–3.84), with ten infants (18.9%) being born with a very low birth weight. The median gestational age was 31 weeks (IQR: 28–37), with fourteen infants (26.4%) being born before 28 weeks and ten patients (18.9%) being born with a birth weight lower than 1000 g. 

Twelve infants (22.6%) were born via vaginal delivery, and twenty-three infants (43.4%) were born via cesarean section; for 18 infants (34%), the mode of delivery was missing/not collected. Forty-nine infants (92.5%) were white, three infants (5.7%) were black/African American, and one (1.9%) had a mixed race. Twenty-nine infants (54.7%) had respiratory distress at birth, and 22 (41.5%) needed orotracheal intubation and mechanical ventilation. Forty-eight infants (90.6%) already had a central venous catheter. 

The demographics of included patients are reported in Table 1.

Fungal infection was microbiologically confirmed in 38 cases (71.7%), with a prevalence of *Candida albicans* in 20 cases (37.7%) and *Candida parapsilosis* in 13 cases (24.5%). In the remaining five patients (9.4%), we identified two cases of *Candida krusei*, one patient with *Candida glabrata*, one patient with *Candida lusitaniae*, and one patient with *Candida tropicalis*. At the start of symptoms of systemic fungal infection, sixteen infants (30.2%) had less than 28 days, twenty-four infants (45.3%) had an age between 28 days and 120 days, and thirteen infants (24.5%) had more than 120 days of life.

The median weight at micafungin administration was 2.19 kg (IQR: 0.75–4.36). Five infants died (9.4%). Three of them were born before 27 weeks GA and had a birth weight lower than 1500 g.

In Table 2, we show micafungin plasma PK characteristics. The mean micafungin clearance was higher in neonates than in older infants (0.036 L/h/kg before 28 days of life versus 0.028 L/h/kg after 120 days). The drug half-life was shorter in neonates than in older patients (13.5 h before 28 days of life versus 14.4 h after 120 days).

**Figure 1:**
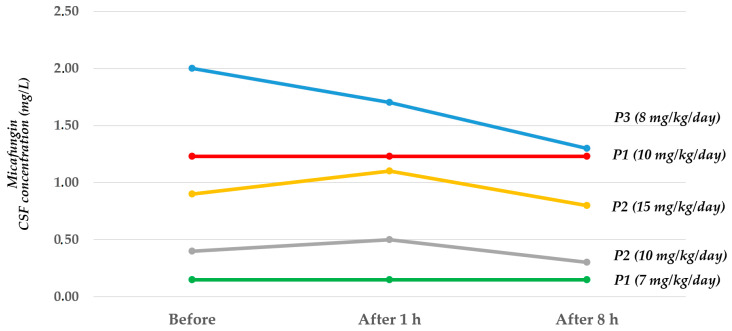
Micafungin concentrations in cerebrospinal fluid before and after different dose infusions in three infants with *Candida* meningitis.

CSF micafungin concentrations were measured in five assessments with three patients: Figure 1 shows how the higher the dose used, the higher the CSF concentration.

We also reported details about CSF and plasma micafungin concentrations (Table 3 and Table 4, respectively) and the CSF/plasma ratios (Table 5) for the same timepoints. 

The mean treatment duration was 16.7 days. The microbiological success of the therapy with micafungin was achieved in the 83.3% of subjects with invasive candidiasis (95% CI = 51.2–97.9), whereas all infants clinically recovered. Only one infant with *C. parapsilosis* required enhanced treatment by introducing amphotericin B liposome.

**Figure 2:**
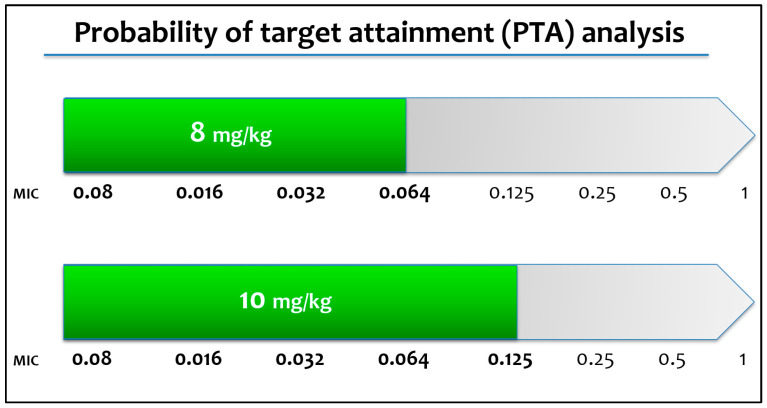
Probability of target attainment calculated for different dosing regimens via Monte Carlo analysis.

The probability of target attainment (PTA) analysis suggests that 4–8 mg/kg may be sufficient for *Candida* spp. with MIC < 0.064 mg/L. Higher doses (10 mg/kg) may be warranted for *Candida* spp. with MIC 0.064 and 0.125 mg/L (Figure 2).

A higher success of mycological eradication was achieved using high doses of micafungin (8–10 mg/kg), even when considering a higher target of the ratio between predicted AUC and the identified minimum inhibitory concentration (MIC) (Table 6).

Thirty-four patients (64.2%) had overall treatment-emergent adverse events (TEAEs); of whom, only three cases of septic shock (5.7%) led to death: they were not drug-related but were due to the underlying severe infection.

Conversely, only three TEAEs (5.7%) were considered to be drug-related but were not serious: one patient (1.9%) had transient hypertransaminasaemia, whereas two (3.8%) had transiently increased levels of gamma-glutamyltransferase.

## 4. Discussion

Via this pooled analysis of the previous two studies, we have demonstrated in critically ill children with invasive candidiasis that micafungin clearance (L/h/kg) increased according to a lower patient’s age and body weight, whereas the half-life was shorter in younger than in older children. Micafungin administered at doses ranging between 8 and 15 mg/kg/day could provide plasma exposures adequate to those required by neonates and infants in cases of invasive fungal infections. The finding of high micafungin values even in neonatal CSF samples of our cohort and the resolution of the meningeal infection suggests that, at doses ranging between 8 and 15 mg/kg/die, micafungin could cross the blood–brain barrier reaching therapeutic levels in CSF in neonates with invasive candidiasis. Such high doses seem necessary in neonates and small critically ill infants, as the drug excretion and clearance do not vary linearly with patient body weight in the age group up to six months [10,11]. The United States Food and Drug Administration recently approved a change in micafungin posology to expand to children younger than 4 months of age, similarly to the labeling in Europe for children of all age groups, including neonates [15,16]. The previously performed studies were used to support the labeling updates in the United States.

In Europe, the label includes a warning of potential hepatotoxicity based on the development of hepatocellular tumors in rats treated for a period of 3 months or more with micafungin at a dose of 150 mg/day, which corresponds to eight times the highest recommended human dose [16,17]. However, our patients showed only transient alterations of transaminases and gamma glutamyl-transferase, which regressed upon the discontinuation of the therapy, and the clinical and laboratory controls carried out following up many of these patients did not cause any concerns from the point of view of liver function.

To date, Amphotericin B deoxycholate or liposomal amphotericin B are recommended as first-line therapies for invasive fungal infections in neonates, but their use can be limited by nephrotoxicity [18]. Fluconazole is recommended as a second-line drug, but hepatotoxicity and azole resistance can limit its use [16,19]; furthermore, fluconazole prophylaxis is recommended against invasive candidiasis in very low and extremely low-birth-weight preterm neonates or in those carriers of central venous catheters [20,21,22,23]. When a fungal infection is suspected in a neonate who is already receiving fluconazole, a different salvage therapy is needed. Furthermore, if a newborn is carrying a central vascular catheter during a fungal infection, the guidelines recommend the removal of the catheter, because conventionally used antifungals do not penetrate the intraluminal biofilm and candidemia persists.

Echinocandins are the most recently developed antifungal drug class and have broad-spectrum efficacy against *Candida* spp., including fluconazole-resistant strains, and some activity against *Aspergillus* spp. [24]. In addition, the echinocandins appear to prevent and reduce biofilm formation in vascular catheters and decrease yeast activity in already-formed biofilms [25,26].

To date, micafungin is the only echinocandin that has been approved by the European Medicines Agency (EMA) for use in neonates [17] and, as explained, recently it has also been approved by the US Food and Drug Administration (FDA) for use in infants less than four months old [23]. 

Although limited, the available data on the efficacy and safety of micafungin in pediatric patients younger than four months old support its use for treating invasive candidiasis in this particular population, in line with the most updated recommendations from the EMA and the FDA [27].

The approval of new drugs, or expanded indications for a previously authorized treatment, are based on considerable evidence of the product’s efficacy and safety established via well-designed clinical studies. Unfortunately, studies about drugs treating uncommon neonatal diseases such as fungal infections are challenging due to the difficulty in enrolling patients [19,28]. A phase 3 randomized, double-blind, active-controlled clinical trial comparing high-dose micafungin to amphotericin B in pediatric patients younger than 4 months initially planned to enroll 225 patients: 150 in the micafungin arm and 75 in the amphotericin B arm. However, due to the difficulty in recruiting patients, the trial was terminated after 2 years with only 20 and 10 subjects enrolled in the micafungin and amphotericin B arms, respectively [29].

Beyond randomized controlled trials, considering that mortality in neonatal intensive care units following *Candida* bloodstream infections is as high as 40%, and neurodevelopmental impairment is common among survivors [28], each piece of additional information about antifungal drugs’ pharmacokinetics is crucial to address a significant unmet need in this vulnerable population. 

In extremely low-birth-weight infants (<1 kg at birth) with invasive candidiasis, the probability of the central nervous system involvement during infection varies from 45% to 63% of cases [30]. Micafungin concentration values in CSF are difficult to obtain if an external shunt has not been placed previously. Moreover, interpreting these values in vivo is not easy, considering that the fungal involvement of the central nervous system in neonatal age is typically hematogenous *Candida* meningoencephalitis (HCME). Hope et al. studied the pharmacokinetics and pharmacodynamics of micafungin in a rabbit model of neonatal HCME and found the highest drug concentrations within the meninges and choroid, whereas micafungin was not reliably detected within the CSF [2]. 

The CSF/plasma ratio shows the penetration of the drug into the cells of the choroid plexuses which in turn have an active metabolism in the secretion of the drug in the CSF, which can change in relation to the current neurological disease. Indeed, the patient with the flat curve of CSF concentrations had multichambered hydrocephalus; so, the diffusion in the cells where the drainage was drawn could be altered. 

We did not observe clinically relevant side effects or changes from the baseline laboratory values; in most patients, liver enzymes and total bilirubin values were in the normal laboratory ranges during the study. One patient (1.9%) had hypertransaminasaemia, whereas two had (3.8%) increased levels of gamma-glutamyltransferase. Transaminases and gamma-glutamyltransferase returned to their normal values after discontinuing the drug. Our results were in line with Schüller’s findings about 19 extremely low-birth-weight neonates treated with a mean micafungin dose of 7.5 ± 2.0 mg/kg micafungin because of suspected or proven invasive candidiasis. They found that micafungin was associated with transiently increased liver enzymes and conjugated bilirubin; alanine transaminase (ALT) levels significantly increased during micafungin therapy, whereas aspartate transaminase (AST) and conjugated bilirubin levels increased during therapy but did not reach statistical significance. However, a sub-analysis also showed that patients who underwent a surgical abdominal intervention had significantly higher AST and ALT levels than infants who did not, indicating that abdominal surgery, associated with prolonged parenteral nutrition, is a crucial confounding factor for micafungin-associated hepatotoxicity in ELBW infants [31].

Indeed, the alteration of hepatic function in our neonates may be due to several other causes (e.g., prematurity, other drugs, and comorbidities), and attributing causality of alterations in liver function tests to micafungin remains difficult and uncertain [27]. Similarly, the EMA issued a black box warning in response to potential hepatocellular tumor growth after three months of micafungin therapy in rats [17]. However, even after using micafungin for a number of years, an attempt is yet to be made to substantiate any potential extrapolation of these results to humans. Early drug discontinuation is recommended in the presence of a significant and persistent elevation of transaminases.

## 5. Conclusions

Micafungin may become even more important for ensuring effective antifungal treatment for pediatric patients with invasive candidiasis who are under the age of four months due to the recent alarming global spread of *Candida auris*, which is frequently resistant to polyenes rather than echinocandins and exhibits high rates of resistance to azoles [27]. Recently, the emergence of the spread of *Candida auris* among preterm neonates has been declared [32,33].

Our findings confirm that using high doses of micafungin (8–15 mg/kg/day) in neonates and infants is safe and allows them to achieve therapeutic success. We observed no serious adverse events related to the therapy of IC with high doses of micafungin.

## Figures and Tables

**Table 1:** Demographics of included patients.

	Mean ± Standard Deviation	Minimum Value	Maximum Value
Birth weight (grams)	1610 ± 940	400	3800
Gestational age (weeks)	31.2 ± 5.3	23.0	40.0
Weight at micafungin infusion (grams)	2620 ± 1380	600	6800
Age at micafungin infusion (months)	2.5 ± 2.1	0.30	8.10

**Table 2:** Micafungin plasma PK characteristics in neonates and young infants up to 6 months of age.

	0 to ≤28 Days (n = 16)	>28 Days ≤120 Days (n = 23)	>120 Days (n = 13)
Clearance (L/h), mean ± SD	0.072 ± 0.066	0.087 ± 0.049	0.109 ± 0.071
Clearance/weight (L/h/kg), mean ± SD	0.036 ± 0.016	0.036 ± 0.010	0.028 ± 0.011
AUC (mg·h/L), mean ± SD	259.0 ± 92.7	236.8 ± 63.9	346.4 ± 145.0
Half-life (h), mean ± SD	13.5 ± 7.6	13.4 ± 4.3	14.4 ± 4.2

**Table 3:** CSF micafungin concentrations in three patients before and after different dose infusions.

	CSF Values before Infusion (mg/L)	CSF Values after 1 h (mg/L)	CSF Values after 8 h (mg/L)
Patient 1 (dose 7 mg/kg/day)	0.15	0.15	0.15
Patient 1 (dose 10 mg/kg/day)	1.23	1.23	1.23
Patient 2 (dose 10 mg/kg/day)	0.40	0.50	0.30
Patient 2 (dose 15 mg/kg/day)	0.90	1.10	0.80
Patient 3 (dose 8 mg/kg/day)	2.00	1.70	1.30

**Table 4:** Plasma micafungin concentrations in three patients before and after different dose infusions.

	Plasma Values before Infusion (mg/L)	Plasma Values after 1 h (mg/L)	Plasma Values after 8 h (mg/L)
Patient 1 (dose 7 mg/kg/day)	2.90	15.60	8.90
Patient 1 (dose 10 mg/kg/day)	3.20	21.70	6.58
Patient 2 (dose 10 mg/kg/day)	6.70	17.40	8.90
Patient 2 (dose 15 mg/kg/day)	5.80	21.90	12.20
Patient 3 (dose 8 mg/kg/day)	9.12	27.00	16.84

**Table 5:** CSF/plasma ratios in three patients before and after different dose infusions.

	CSF/Plasma Ratio before Infusion	CSF/Plasma Ratio after 1 h	CSF/Plasma Ratio after 8 h
Patient 1 (dose 7 mg/kg/day)	0.05	0.01	0.02
Patient 1 (dose 10 mg/kg/day)	0.38	0.06	0.19
Patient 2 (dose 10 mg/kg/day)	0.06	0.03	0.03
Patient 2 (dose 15 mg/kg/day)	0.16	0.05	0.07
Patient 3 (dose 8 mg/kg/day)	0.22	0.06	0.08

**Table 6:** Microbiological success according to the different doses of micafungin, the identified minimum inhibitory concentration (MIC), and the AUC/MIC ratio.

	Target	AUC/MIC ≥ 3000			
	Dose (mg/kg)	2	4	8	10
**MIC (mg/L)**	0.008	88.8%	97.3%	99.7%	99.9%
	0.016	56.3%	88.8%	97.3%	98.6%
	0.032	11.2%	56.3%	88.8%	92.9%
	0.064	0.3 %	11.2%	56.3%	70.7%
	0.125	0.0%	0.3%	11.2%	23.5%
	0.25	0.0%	0.0%	0.3%	1.3%
	0.5	0.0%	0.0%	0.0%	0.0%
	1	0.0%	0.0%	0.0%	0.0%
	**Target**	**AUC/MIC ≥ 2000**			
	**Dose (mg/kg)**	**2**	**4**	**8**	**10**
**MIC (mg/L)**	0.008	94.9%	99.1%	99.9%	100.0%
	0.016	79.7%	94.9%	99.1%	99.5%
	0.032	34.2%	79.7%	94.9%	96.9%
	0.064	3.4%	34.2%	79.7%	87.0%
	0.125	0.0%	3.8%	35.9%	53.3%
	0.25	0.0%	0.0%	3.8%	9.9%
	0.5	0.0%	0.0%	0.0%	0.2%
	1	0.0%	0.0%	0.0%	0.0%
	**Target**	**AUC/MIC ≥ 1000**			
	**Dose (mg/kg)**	**2**	**4**	**8**	**10**
**MIC (mg/L)**	0.008	99.1%	99.9%	100.0%	100.0%
	0.016	94.9%	99.1%	99.9%	100.0%
	0.032	79.7%	94.9%	99.1%	99.5%
	0.064	34.2%	79.7%	94.9%	96.9%
	0.125	3.8%	35.9%	79.7%	87.8%
	0.25	3.8%	3.8%	35.9%	53.3%
	0.5	0.0%	0.0%	3.8%	9.9%
	1	0.0%	0.0%	0.0%	0.0%

## Data Availability

Researchers may request access to anonymized participant level data, trial level data, and protocols from Astellas sponsored clinical trials at www.clinicalstudydatarequest.com, (accessed on 1 February 2023). For the Astellas criteria on data sharing, see https://clinicalstudydatarequest.com/Study-Sponsors/Study-Sponsors-Astellas.aspx, (accessed on 1 February 2023).
